# Peer Review of “Social Media Polarization and Echo Chambers in the Context of COVID-19: Case Study”

**DOI:** 10.2196/32268

**Published:** 2021-08-05

**Authors:** Shana Gadarian

**Affiliations:** 1 Department of Political Science Maxwell School of Citizenship and Public Affairs Syracuse University Syracuse, NY United States

**Keywords:** social media, opinion, COVID-19, case study, polarization, communication, Twitter, echo chamber

*This is a peer-review report submitted for the paper “Social Media Polarization and Echo Chambers in the Context of COVID-19: Case Study.”*

## Round 1 Review

### General Comments

This paper [1] studies the polarization of COVID-19 discourse on Twitter using natural language processing (the Retweet-BERT method).

The authors are interested in whether partisan users interact mostly with like-minded partisans and how polarized influential users are. They estimate the partisan nature of users/accounts by who a user retweets—with the assumption that users will follow people who they agree with. The concern here is that in estimating ideology from retweets and then looking at echo chambers, aren’t the authors building endogeneity into the measures? The networks one belongs to and follows are certainly a measure of something, but it is not clear that this is separate from the information environment or potential echo chamber. Can the authors theoretically separate the network one belongs to from the sharing of information if retweeting is the basis for the ideology of the respondent? The methods of finding ground truth using hashtags and media retweets seem more appropriate than the method that the authors propose given the theoretical similarity between a user’s network and what they tweet or share.

It would also be helpful to have additional theoretical justification for the decision to bin the polarity scores due to the left-skewed nature of the left-leaning seed users. Are the findings robust for thinking about the online space compared to a benchmark of partisanship from national surveys rather than compared to only people online? In other words, what seems like “polarity” online might be extreme or might be only a subset of the entire ideological space in the United States, and it is not clear whether the authors are interested in only Twitter users or want to say something about how people online generally share political information.

The article says that it is about COVID-19 information but there is very little discussion of the content of that information and why or how the authors might expect COVID-19 information to be shared differently than other information. Is this a demonstration of this tool in a particular time period or is there something about COVID-19 information that would make it more likely to be shared? The evidence that right-leaning users retweet right-leaning accounts is not necessarily an issue for polarization or for public health unless the accounts have different information from public health experts or misinformation. Can the authors speak to that?

## References

[1] Jiang J, Ren X, Ferrara E (2021). Social Media Polarization and Echo Chambers in the Context of COVID-19: Case Study. JMIRx Med.

